# Intravenous Colistin Monotherapy versus Combination Therapy against Carbapenem-Resistant Gram-Negative Bacteria Infections: Meta-Analysis of Randomized Controlled Trials

**DOI:** 10.3390/jcm7080208

**Published:** 2018-08-10

**Authors:** I-Ling Cheng, Yu-Hung Chen, Chih-Cheng Lai, Hung-Jen Tang

**Affiliations:** 1 Department of Pharmacy, Chi Mei Medical Center, Liouying, Tainan 73657, Taiwan; bokey1010@gmail.com (I.-L.C.); her0windqoo@gmail.com (Y.-H.C.); 2 Department of Intensive Care Medicine Chi Mei Medical Center, Liouying, Tainan 73657, Taiwan; dtmed141@gmail.com; 3 Department of Medicine, Chi Mei Medical Center, Tainan 71004, Taiwan

**Keywords:** colistin, monotherapy, combination therapy, carbapenem-resistant bacteria

## Abstract

This meta-analysis aims to compare intravenous colistin monotherapy and colistin-based combination therapy against carbapenem-resistant gram-negative bacteria (GNB) infections. PubMed, Embase, and Cochrane databases were searched up to July 2018. Only randomized controlled trials (RCTs) evaluating colistin alone and colistin-based combination therapy in the treatment of carbapenem-resistant GNB infections were included. The primary outcome was all-cause mortality. Five RCTs including 791 patients were included. Overall, colistin monotherapy was associated with a risk ratio (RR) of 1.03 (95% confidence interval (CI), 0.89–1.20, I^2^ = 0%) for all-cause mortality compared with colistin-based combination therapy. The non-significant difference was also detected in infection-related mortality (RR, 1.23, 95% CI, 0.91–1.67, I^2^ = 0%) and microbiologic response (RR, 0.86, 95% CI, 0.72–1.04, I^2^ = 62%). In addition, no significant difference was observed in the subgroup analysis—high or low dose, with or without a loading dose, carbapenem-resistant *Acinetobacter baumannii* infections, and in combination with rifampicin. Finally, colistin monotherapy was not associated with lower nephrotoxicity than colistin combination therapy (RR, 0.98; 95% CI, 0.84–1.21, I^2^ = 0%). Based on the analysis of the five RCTs, no differences were found between colistin monotherapy and colistin-based combination therapy against carbapenem-resistant GNB infections, especially for *A. baumannii* infections.

## 1. Introduction

Carbapenem-resistance among gram-negative bacteria (GNB), including *Acinetobacter baumannii*, *Pseudomonas aeruginosa*, and Enterobacteriaceae, has significantly increased all over the world and poses a significant threat to public health [1,2,3]. Most importantly, the infections, such as ventilator-associated pneumonia (VAP), bloodstream infection (BSI), and complicated intra-abdominal infection (IAI) caused by carbapenem-resistant bacteria, are associated with high morbidity and mortality [4,5,6]. However, infections caused by these carbapenem-resistant bacteria are difficult to treat due to compromised treatment options.

Although colistin is an old antibiotic, it remains as one of the limitedly available options against carbapenem-resistant bacteria. In addition, several ways including loading dose [7], higher maintenance dose [8], adjunct local administration [9], and combination therapy are proposed to enhance its activity. Regarding combination therapy, several in vitro studies [10,11,12] have shown the synergy or additive effects of colistin plus sulbactam, fosfomycin, tigecycline, or carbapenem. However, clinical studies did not show consistent results regarding the synergistic effect of colistin-based combination therapy. To unravel this controversial issue, two meta-analyses were conducted by Zusman et al. [13] in 2017 and Vardakaset et al. [14] in 2018, respectively. In these two meta-analyses, most of the enrolled studies were retrospective observational studies, and only three randomized controlled trials (RCTs) [15,16,17] were enrolled. Thus, their conclusions [13,14] were based on low-quality evidence. Recently, two more RCTs [18,19] compared the effects of colistin monotherapy and combination therapy against carbapenem-resistant gram-negative bacteria infections. Therefore, we performed a comprehensive and updated meta-analysis of these five RCTs to provide better evidence of the efficacy of colistin monotherapy and colistin-based combination therapy on treating carbapenem-resistant bacteria infections.

## 2. Methods

### 2.1. Study Search and Selection

All clinical studies were identified by a systematic review of the literature in the PubMed, Embase, and Cochrane databases until July 2018 using the following search terms: “colistin or polymyxin”, “gram negative bacteria or *Acinetobacter baumannii* or *Klebsiella pneumoniae* or *Pseudomonas aeruginosa* or Enterobacteriaceae”, and “prospective or randomized” (Appendix A). Only randomized controlled trials were considered eligible for inclusion and only if they directly compared the clinical effectiveness of colistin monotherapy and colistin-based combination antimicrobial agents in the treatment of documented adult patients with carbapenem-resistant bacteria. We did not include studies with inhaled colistin therapy. Studies were excluded if they focused on in vitro activity or pharmacokinetic-pharmacodynamic assessment. The articles of all languages of publication could be included. Two reviewers (I.-L.C. and Y.-H.C.) searched and examined publications independently to avoid bias. When they had disagreement, a third author (C.-C.L.) resolved the issue in time. The following data, including year of publication, study place, type of infections, patients’ demographic characteristics, the dosage of colistin including loading dose and combined antimicrobial regimens, microbiological outcomes, and mortality, were extracted from every included study.

### 2.2. Definitions and Outcome

The primary outcome was all-cause mortality at any timeframe. However, if the data could be provided by the individual studies, 28- or 30-day mortality was included in the analyses. Secondary outcomes included infection-related mortality, microbiologic response rate, and the nephrotoxicity. The high dose of colistin used was defined as the mean/median colistin dose or the administered dose reported in the study of >6 million international units (MIU), as previously described [14].

### 2.3. Data Analysis

The quality of enrolled RCTs and the risk of bias was assessed using the Cochrane risk of bias assessment tool [20]. We used Review Manager version 5.2 (The Nordic Cochrane Centre, The Cochrane Collaboration, Copenhagen, Denmark) to perform statistical analysis. The degree of heterogeneity was evaluated with Q statistics generated from the χ^2^ test, and I^2^ measure was used to assess the proportion of statistical heterogeneity. Heterogeneity was defined as significant when the *p*-value was less than 0.10 or I^2^ more than 50%. The fixed-effects model was used when the data was homogenous, and the random-effects model was used when they were heterogenous. The pooled risk ratio (RR) and 95% confidence interval (CI) was calculated for outcome analysis. Subgroup and sensitivity analyses were performed according to dose of colistin, the combination regimen, and causative pathogen.

## 3. Results

### 3.1. Study Selection and Characteristics

**Figure 1:**
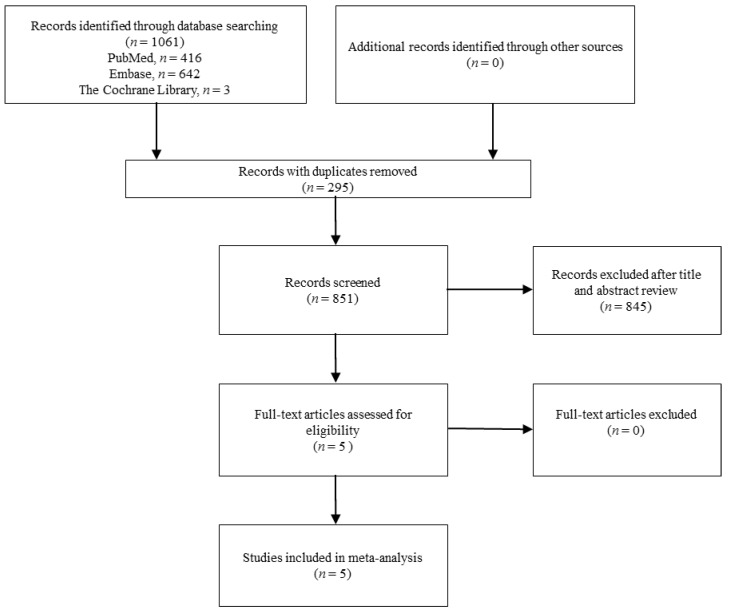
Flow diagram of the study selection process.

**Figure 2:**
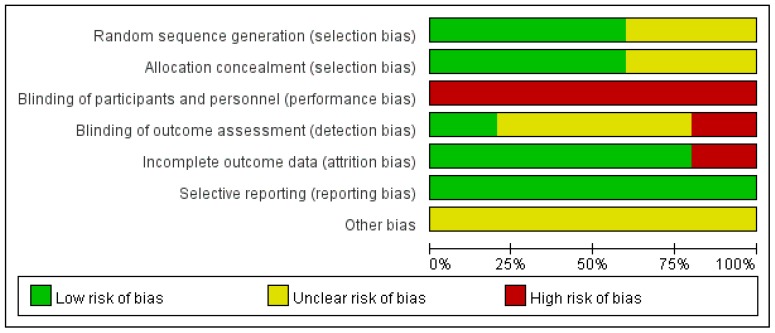
Summary of risk of bias.

**Figure 3:**
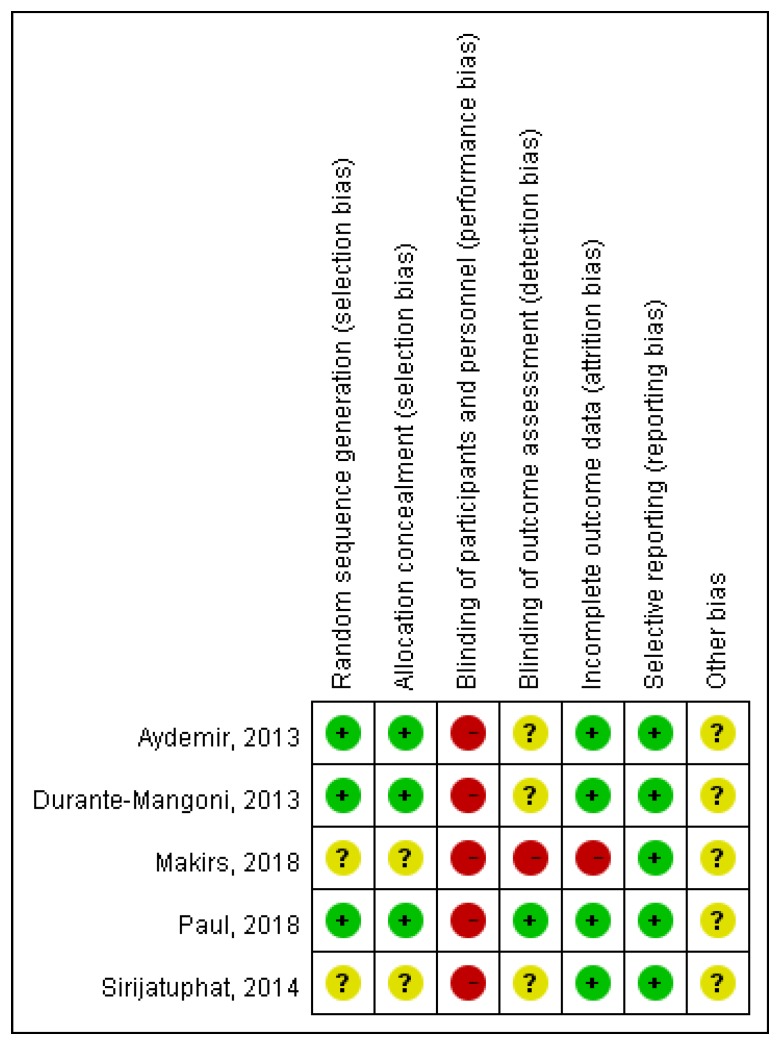
Risk of bias per study and domain.

The search program yielded 1061 references, including 416 from PubMed, 642 from Embase, and 3 from the Cochrane database. Then 851 articles were screened after excluding 295 duplicated articles. Finally, a total of five RCTs [15,16,17,18,19] fulfilling the inclusion criteria were included in this meta-analysis (Figure 1). All of the studies were designed to investigate the outcome of patients with colistin monotherapy or colistin-based combination regimen (Table 1) [15,16,17,18,19]. The number of patients enrolled in each study ranged from 39 to 406. The mean age of patients ranged from 56.8 to 68.3 years, and 14.0–49.8% patients had chronic kidney diseases among these five studies. Only one trial [19] was a multinational study, and all studies [15,16,17,18,19] were performed in Europe or Asia. Despite two studies [17] focused on extensively drug resistant (XDR), *A. baumannii*, which was defined as resistance to carbapenem and all other antibiotics except colistin, all of these *A. baumannii* isolates were resistant to carbapenem. Three studies [15,17,18] were conducted in an intensive care unit (ICU). The antibiotic combination regimens included rifampicin (2 trials) [15,17], fosfomycin (1) [16], meropenem (1) [19], and ampicillin-sulbactam (1) [18]. A high dose of colistin was used in four studies [15,16,18,19], and only one study [17] used a low dose of colistin. A loading dose of colistin was used in one study [19]. The total number of patients in the included RCTs was 791. Pneumonia, including VAP and hospital-acquired pneumonia (HAP), was the most common type of infection, followed by bloodstream infections. All of the studies were open label, and most of the domains were classified as low risk of bias, except performance bias—the blinding of participants and personnel (Figure 2 and Figure 3).

### 3.2. Clinical Outcomes and Microbiological Response

**Figure 4:**
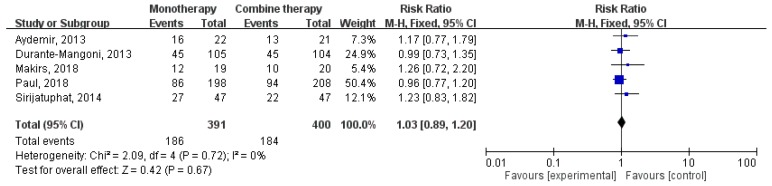
Colistin monotherapy versus colistin-based combination therapy, all-cause mortality. M-H, Mantel-Haenszel.

**Figure 5:**
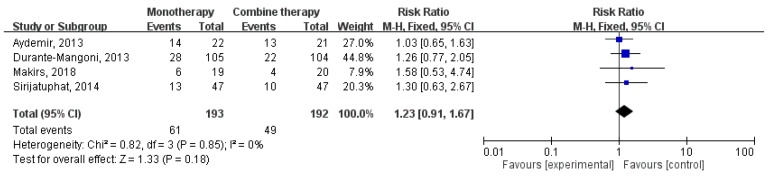
Colistin monotherapy versus colistin-based combination therapy, infection-related mortality. M-H, Mantel-Haenszel.

**Figure 6:**
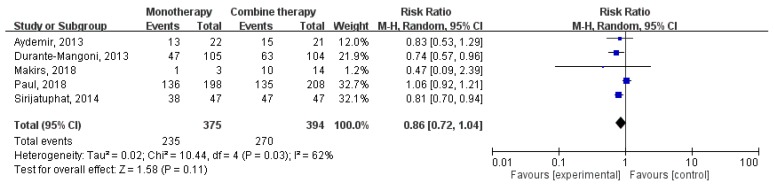
Colistin monotherapy versus colistin-based combination therapy, microbiologic response. M-H, Mantel-Haenszel.

Overall, colistin monotherapy was not associated with higher mortality than colistin combination therapy (RR, 1.03; 95% CI, 0.89–1.20; I^2^ = 0%; Figure 4). Sensitivity analysis after deleting an individual study each time to reflect the influence of the single dataset on the pooled RR showed similar findings. Four studies [15,16,17,18] had the data of infection-related mortality, and colistin monotherapy was not associated with higher infection-related mortality (RR, 1.23; 95% CI, 0.91–1.67; I^2^ = 0%; Figure 5). In addition, colistin monotherapy was not associated with lower microbiologic response (RR, 0.86; 95% CI, 0.72–1.04; I^2^ = 62%; Figure 6).

### 3.3. Subgroup Analysis

Two studies [15,17] with 252 patients enrolled compared colistin monotherapy and a colistin–rifampicin combination, colistin monotherapy was not associated with higher mortality than colistin combination therapy (RR, 1.00; 95% CI, 0.80–1.34; I^2^ = 0%). In addition, no significant difference regarding mortality was found between colistin monotherapy and colistin-based combination therapy in terms of the usage of colistin (loading vs no loading, and high dose vs low dose).

For the 697 patients with carbapenem-resistant *A. baumannii* infections, colistin monotherapy was not associated with higher mortality than colistin-based combination therapy (RR, 1.00; 95% CI, 0.86–1.16; I^2^ = 0%; Figure 5). For the 94 patients with carbapenem-resistant GNB other than *A. baumannii*, colistin monotherapy was not associated with higher mortality than colistin-based combination therapy (RR, 1.60; 95% CI, 0.81–3.15).

### 3.4. Nephrotoxicity

Three studies report the risk of nephrotoxicity according to risk, injury, failure, loss, end stage renal disease (RIFLE) criteria. Colistin monotherapy was not associated with lower nephrotoxicity than colistin combination therapy (RR, 0.98; 95% CI, 0.84–1.21; I^2^ = 0%). 

## 4. Discussion

This analysis based on five RCTs with 791 patients showed that the mortality of carbapenem-resistant GNB infections did not change significantly between colistin-based combination therapy and colistin monotherapy. Similar findings were also noted in other comparisons, such as microbiologic response and infection-related mortality. In addition, this result was not affected by the dose of colistin or combined antibiotic regimens. In Zusman’s analysis of seven observational studies with 537 patients, colistin monotherapy was associated with an unadjusted odds ratio (OR) of 1.58 (95% CI, 1.03–2.42) for mortality compared with a colistin–carbapenem combination [13]. In addition, colistin monotherapy was found to be associated with higher mortality than colistin–tigecycline, –aminoglycosides, or –fosfomycin combination therapy (uOR, 1.57, 95% CI, 1.06–2.32) based on 10 observational studies and one RCT [13]. In another analysis including 29 observation studies and three RCTs by Vardakas et al. [14], colistin combination therapy was not associated with lower mortality than colistin monotherapy (RR, 0.91; 95% CI, 0.81–1.02). In contrast to the above two analyses that lack enough data from RCTs, the present meta-analysis only enrolled RCTs and used more updated and larger data from two RCTs [18,19] in 2018, especially Paul et al.’s study, which enrolled 406 patients. Except one RCT involving only 39 patients conducted by Makris et al. [18], all of the other four RCTs showed consistent results. Therefore, the level of evidence in this meta-analysis is more solid than that of the previous two analyses [13,14].

Colistin is an important antimicrobial agent for the treatment of carbapenem-resistant or extensively drug resistant *A. baumannii* infections [21,22]. In this meta-analysis, four out of the five studies [15,16,17,18] focused on carbapenem-resistant *A. baumannii*, and about 312 (77%) out of 406 cases in another study [19] were caused by carbapenem-resistant *A. baumannii* as well. In the subgroup analysis of carbapenem-resistant *A. baumannii* infections, we found that colistin combination therapy was not associated with lower mortality rate than monotherapy. This finding is in concordance with Chen et al.’s analysis [23] that the clinical response and in-hospital mortality did not differ between colistin monotherapy and colistin-based combination therapy (clinical response—OR, 1.37, 95% CI, 0.86–2.19, *p* = 0.18; mortality—RR, 0.93, 95% CI, 0.74–1.17, *p* = 0.54). However, only two RCTs were included in Chen’s meta-analysis. By our findings based on five RCTs, the issue that colistin-based combination was not superior to monotherapy for carbapenem-resistant *A. baumannii* infections becomes clearer than previously reported [23].

In this meta-analysis, two RCTs [15,17] compared the effects of colistin monotherapy and colistin–rifampicin combination therapy. We did not find any statistical significance in terms of mortality and microbiologic response. It indicated that colistin monotherapy has a similar treatment outcome to colistin–rifampicin combination therapy. However, further studies are required to confirm this finding.

Three RCTs [16,17,19] in this meta-analysis reported the risk of acute kidney injury, and we found that combination therapy was not associated with a higher risk of nephrotoxicity than monotherapy. However, the antibiotic combination regimen differed in these three RCTs—rifampicin, fosfomycin, and meropenem was used in each study. Thus, we cannot make solid conclusion based our findings. We still need more studies to clarify this issue.

This meta-analysis has one major strength. Only RCTs were included, so the risk of bias should be minimized, and the level of evidence could be strong. However, this meta-analysis also has several limitations. First, the differences in study subjects, disease severity, setting, and type of infections between individual studies made the study population heterogeneous. Second, the number of included RCTs and the study subjects are limited, and the colistin-based combined therapy only included rifampicin, fosfomycin, meropenem, and sulbactam. A further large-scale study with various colistin-based combination regimens is warranted.

In conclusion, based on the analysis of five RCTs, no differences were found in the effects of colistin monotherapy and colistin-based combination therapy against carbapenem-resistant GNB infections. However, additional studies are still needed to evaluate the effect of different colistin-based combination regimens compared with colistin monotherapy in carbapenem-resistant GNB infections, especially for *A. baumannii* infections.

## Figures and Tables

**Table 1:** Characteristics of included studies.

Author/Publication Year	Study Year	Study Site	Bacteria	Polymicrobial	Setting	Infection Type (%)	Usage of IV Colistin Dose	No. of Polymyxin	No. of Combination with
Aydemir, 2013	2011–2012	Turkey	Carbapenem-resistant *A. baumannii*	No	ICU	VAP (100)	300 mg colistin based activity/day, t.id. (9 MIU per day)	Colistin (22)	Rifampicin (21)
Durante-Mangoni, 2013	2010–2011	Italy	Extensive-drug resistant *A. baumannii*	Yes	ICU	VAP (69), BSI (20), HAP (9), cIAI (2)	2 MIU every 8 h	Colistin (105)	Rifampicin (104)
Sirijatuphat, 2014	2010–2011	Thailand	Carbapenem-resistant *A. baumannii*	Yes	ICU and ward	Pneumonia (76.6), BSI (5.4), UTI (5.4), IAI (6.4), SSTI (3.2), CNSI (1.0), other (2.1)	5 mg colistin based activity/kg/day (9 MIU per day)	Colistin (47)	Fosfomycin (47)
Paul, 2018	2013–2016	Israel, Greece, Italy	Carbapenem-resistant gram-negative bacteria, including *A. baumannii*, Enterobacteriaceae, Pseudomonas, and others	No	ICU and ward	VAP/HAP (44.8), BSI (42.6), UTI (6.4), *p*VAP (6.2)	9 MIU loading, followed by 4.5 MIU every 12 h	Colistin (198)	Meropenem (208)
Makirs, 2018	-	Greece	Carbapenem-resistant *A. baumannii*	No	ICU	VAP (100)	3 MIU t.i.d.	Colistin (19)	Ampicillin-sulbactam (20)

Abbreviations: IV, intravenous; ICU, intensive care unit; MIU, million international units; VAP, ventilator-associated pneumonia; BSI; bloodstream infection; HAP, hospital-acquired pneumonia; cIAI, complicated intra-abdominal infection; UTI, urinary tract infection; SSTI, skin and soft tissue infection; CNSI, central nervous system infection; t.i.d, three times per day.

## Appendix A. List of Terms of the Search Strategy

**PubMed**

1. “colistin” [Mesh]
2. “colistin” [All Fields]
3. “polymyxins“ [All Fields]
4. 1 OR 2 OR 3
5. “gram-negative bacteria“ [MeSH]
6. “acinetobacter baumannii“ [All Fields]
7. “klebsiella pneumoniae“ [MeSH Terms]
8. “klebs a pneumoniae“ [All Fields]
9. “pseudomonas aeruginosa“ [MeSH Terms]
10. “pseudomonas aeruginosa“ [All Fields]
11. “enterobacteriaceae“ [MeSH Terms]
12. 5 OR 6 OR 7 OR 8 OR9 OR 10 OR 11
13. “randomized“ [All Fields]
14. “randomised“ [All Fields]
15. “longitudinal studies“ [MeSH Terms]
16. “longitudinal studies“ [All Fields]
17. “prospective“ [All Fields]
18. 13 OR 14 OR 15 OR 16 OR 17
19. 4 AND 12 AND 18

**Embase**

1. colistin
2. polymyxin
3. 1 OR 2
4. gram negative bacteria
5. acinetobacter baumannii
6. klebsiella pneumonia
7. pseudomonas aeruginosa
8. enterobacteriaceae
9. 4 OR 5 OR 6 OR 7 OR 8
10. randomized
11. prospective
12. 13 OR 14
13. 3 AND 9 AND 12

**Cochrane**

1. MeSH descriptor colistin explode all trees
2. MeSH descriptor polymyxin explode all trees
3. 1 OR 2
